# Carotenoid Supplementation for Alleviating the Symptoms of Alzheimer’s Disease

**DOI:** 10.3390/ijms25168982

**Published:** 2024-08-18

**Authors:** Jolanta Flieger, Alicja Forma, Wojciech Flieger, Michał Flieger, Piotr J. Gawlik, Eliasz Dzierżyński, Ryszard Maciejewski, Grzegorz Teresiński, Jacek Baj

**Affiliations:** 1 Department of Analytical Chemistry, Medical University of Lublin, Chodźki 4a, 20-093 Lublin, Poland; 2 Department of Forensic Medicine, Medical University of Lublin, ul. Jaczewskiego 8b, 20-090 Lublin, Poland; aforma@onet.pl (A.F.); michalflieeeger@gmail.com (M.F.); grzegorzteresinski@umlub.pl (G.T.); 3 Department of Plastic Surgery, St. John’s Cancer Center, ul. Jaczewskiego 7, 20-090 Lublin, Poland; wwoj24@wp.pl (W.F.);; 4 Institute of Health Sciences, John Paul II Catholic University of Lublin, Konstantynów 1 H, 20-708 Lublin, Poland; ryszard.maciejewski@kul.pl; 5 Department of Correct, Clinical and Imaging Anatomy, Medical University of Lublin, ul. Jaczewskiego 4, 20-090 Lublin, Poland; jacek.baj@umlub.pl

**Keywords:** Alzheimer’s disease, carotenoids, brain, memory loss, dementia, cognitive dysfunction, neuroprotection, natural products

## Abstract

Alzheimer’s disease (AD) is characterized by, among other things, dementia and a decline in cognitive performance. In AD, dementia has neurodegenerative features and starts with mild cognitive impairment (MCI). Research indicates that apoptosis and neuronal loss occur in AD, in which oxidative stress plays an important role. Therefore, reducing oxidative stress with antioxidants is a natural strategy to prevent and slow down the progression of AD. Carotenoids are natural pigments commonly found in fruits and vegetables. They include lipophilic carotenes, such as lycopene, α- and β-carotenes, and more polar xanthophylls, for example, lutein, zeaxanthin, canthaxanthin, and β-cryptoxanthin. Carotenoids can cross the blood–brain barrier (BBB) and scavenge free radicals, especially singlet oxygen, which helps prevent the peroxidation of lipids abundant in the brain. As a result, carotenoids have neuroprotective potential. Numerous in vivo and in vitro studies, as well as randomized controlled trials, have mostly confirmed that carotenoids can help prevent neurodegeneration and alleviate cognitive impairment in AD. While carotenoids have not been officially approved as an AD therapy, they are indicated in the diet recommended for AD, including the consumption of products rich in carotenoids. This review summarizes the latest research findings supporting the potential use of carotenoids in preventing and alleviating AD symptoms. A literature review suggests that a diet rich in carotenoids should be promoted to avoid cognitive decline in AD. One of the goals of the food industry should be to encourage the enrichment of food products with functional substances, such as carotenoids, which may reduce the risk of neurodegenerative diseases.

## 1. Introduction

Dementia and cognitive dysfunction are the results of neurological disorders associated with aging, but also with the development of AD. Due to the lack of effective therapy, effective interventions are needed to slow down neurodegenerative processes. In recent years, much attention has been paid to a proper diet and introducing so-called nutraceuticals, including phytochemical compounds such as flavonoids, carotenoids, and polyphenolic acids [1,2,3,4,5,6,7]. They help fight oxidative stress, have anti-inflammatory properties, and regulate mitochondrial dysfunction and the dysbiosis of intestinal microflora. AD is a neurodegenerative disease (NDD) that, according to the World Health Organization (WHO), affects millions of people worldwide [8]. It is estimated that by 2030, the number of AD patients will exceed 80 million, and by 2050, this number may double [9].

AD has many characteristic symptoms, the most common of which are memory loss, the deterioration of cognitive functions, the deterioration of motor coordination, mood swings, speech problems, decreased executive ability, etc. [10,11]. Typical features of AD are the presence of extracellular amyloid-beta (Aβ) plaques and neurofibrillary tangles formed by the aggregation of hyperphosphorylated tau protein located intracellularly mainly in the hippocampus and cerebral cortex [12].

The development of AD is associated with several risk factors, including genetic predisposition, exposure to environmental toxins and metals, brain injuries, infectious diseases, decreased immunity, and vascular diseases [13,14]. Recently, there has been in-creased attention given to the impact of elevated levels of pro-inflammatory cytokines, chemokines, interleukin (IL) [15,16], oxidative stress, metabolic changes, mitochondrial dysfunction, neuronal apoptosis, and synaptic neurotransmission disorders [17,18] on the development of AD. Memory and learning processes involve serotonin (5-HT) and cholinergic neurons, which synthesize acetylcholine (ACh) [19], especially in the hippo-campus and cortical area [20,21,22,23]. In AD, reductions in 5-HT [23], the loss of cholinergic neurons, the increased expression of the gamma-aminobutyric acid (GABA) receptor [24], and the disruption of glutamatergic neurotransmission [25] are observed.

The characteristic structural brain abnormalities and mental impairment associated with AD are linked to high levels of reactive oxygen species (ROS) and neuronal apoptosis, which may indicate low levels of antioxidants [26]. Research has confirmed an increase in lipid peroxidation in cerebral cortex tissue samples taken during autopsies of AD patients [27]. The prevention of Alzheimer’s disease (AD) can involve natural methods that focus on introducing a wide range of biologically active substances into the diet, such as flavonoids, phenolic acids, and carotenoids. The therapeutic benefits of using these nutraceuticals have been supported by in vivo and in vitro studies. In recent years, in addition to carotenoids, much attention has been paid to other plant bioactive compounds of nutraceutical nature, such as glucosinolates, polyphenols, terpenes, sulfide compounds, phytosterols, and phytoestrogens. In addition to their nutritional value, they have therapeutic effects, such as antioxidant, anti-inflammatory, and neuroprotective activity [28,29]. 

The effectiveness of nutraceuticals has been confirmed in the treatment of, among others, cancer, diabetes, allergies, obesity, and cardiovascular and neurological diseases [30]. The most popular nutraceuticals are polyphenolic compounds, including phenolic acids, flavonoids, anthocyanins, stilbenes, and lignans. The content of polyphenols in food can be found in a comprehensive online database, e.g., Phenol-Explorer [31]. Kelsey et al. [32] described in their review the neuroprotective effects of selected nutraceuticals, such as epigallocatechin gallate (EGCG), quercetin, curcumin, resveratrol, rosmarinic acid or carnosic acid, and organosulfur compounds, including isothiocyanate, L-sulforaphane, and allicin thiosulfonate.

Over 1.5 thousand herbal preparations of a nutraceutical nature have been registered in the European Union. The market overview of these products is published on the ReportLinker website (https://www.reportlinker.com, accessed on 8 March 2024). The value of the nutraceutical market is still growing. Currently, it is about USD 30 billion per year [33]. In the case of neurodegenerative diseases, the effectiveness of nutraceuticals such as curcumin, α-lipoic acid, astaxanthin, coenzyme Q10 (ubiquinone), L-sulforaphane (isothiocyanate compound), tert-butylhydroquinone, blueberry, resveratrol, carnosic acid, eugenol, emodin (3-methyl-1,6,8-trihydroxyanthraquinone), rosmarinic acid, old garlic extract, anthocyanins, epigallocatechin-3-gallate (green tea flavonoid), mustard oil glycoside, retinoic acid, vitamin D, vitamin E, polyunsaturated omega-3 fatty acid, apigenin, soy isoflavones, and isoflavones has been demonstrated [34]. However, there are also risks associated with the use of herbal preparations in the treatment of neurodegenerative diseases. Plant dietary supplements may contain various contaminants such as mycotoxins, heavy metals, and pesticides [35]. Natural carotenoid pigments, including β-carotene, lycopene, lutein, zeaxanthin, astaxanthin, fucoxanthin, crocin, and others, have potential in the treatment of AD. The development of effective pharmaceutical formulations must address the limitations of poor bioavailability and stability. Therefore, ongoing efforts are concentrated on developing carotenoid nanosystems for treating AD. An up-to-date review of research on various carotenoid delivery nanosystems was published by Su et al. [36].

We searched the PubMed database for articles on using carotenoids in prevention and neuroprotection over the last five years [9,36,37,38,39,40,41,42,43,44,45,46,47,48,49,50]. In review articles published in the previous year, the authors focused on the impact of diet and dietary carotenoids on various neurodegenerative diseases [51,52,53], as well as the antioxidant properties of carotenoids [54]. Reviews have also concerned the use of different phytochemicals, including carotenoids, in Alzheimer’s disease (AD) therapy [9,55,56,57,58], the use of a single natural chemical compound in AD therapy [46], or the use of a selected substance from the carotenoid group in the treatment of many neurodegenerative diseases [59,60,61]. In 2022, Batool et al. [37] published a comprehensive evidence-based review on the use of natural carotenoids in the treatment of Alzheimer’s disease, focusing specifically on astaxanthin, fucoxanthin, macular carotenoids, and crocin. In our article, we summarized the latest research on the potential use of carotenoids (carotenes and xanthophylls) in the prevention and treatment of AD. We have expanded the discussed group of carotenoids to include lycopene, β-carotene, lutein, zeaxanthin, astaxanthin, fucoxanthin, β-cryptoxanthin, crocin, and crocetin. In addition, we have presented therapies proposed in recent years for the alleviation of AD symptoms based on carotenoid supplementation.

## 2. Pathogenesis of AD

The histopathological manifestations of Alzheimer’s disease (AD) include the aggregation of Aβ (amyloid beta) plaques and the hyperphosphorylation of tau protein [62]. This leads to abnormal folding and the formation of toxic and insoluble neurofibrillary tangles, particularly in the hippocampus and cerebral cortex. The activation of microglia due to inflammation of the nervous system is believed to be a possible mechanism in the pathogenesis of AD [63].

There is evidence suggesting that oxidative stress also plays a role in the development of AD [64,65,66,67,68]. The brain is vulnerable to oxidative stress due to its high oxygen demand and the presence of fats. Oxidative stress causes lipid peroxidation, leading to the inactivation of enzymes, reduced cell membrane fluidity, and the inactivation of receptors and ion channels. This stress also disrupts neurotransmitter transmission and neuronal function, ultimately having a destructive effect on brain activity.

The increase in reactive oxygen species (ROS) or reactive nitrogen species (RNS) [68] resulting from increased production, insufficient antioxidants, or redox imbalance triggers a cascade of events. These events range from damaging the structure of macromolecules, proteins, lipids, and DNA to the aggregation of Aβ plaques [69,70], as well as the aggregation and phosphorylation of tau [71], and disruptions in neurotransmission, all culminating in neuronal apoptosis [71,72,73,74]. Studies suggest that ROS induces apoptosis through NF-κB, an oxidative-stress-responsive transcription factor [75].

Neurotoxic Aβ plaques are formed from the 120 kDa transmembrane amyloid precursor protein (APP) due to amyloidogenic and non-amyloidogenic proteolytic degradation processes influenced by α-, β-, and γ-secretases [76]. The β-secretase digestive pathway predominates in neurons, while α-secretases are active in non-neuronal cells [77]. In addition to the classic secretase-dependent mechanisms of APP degradation, there are also less known secretase-independent pathways. Caspases, particularly caspase-3, may also participate in the secretase-independent degradation of APP [76]. They likely play a minor role in the formation of amyloidogenic products and the development of AD [78,79].

Aβ peptides are released in trace picomolar amounts in the physiological state as a product of the metabolism of various cells [80]. Such a low concentration of extracellular soluble Aβ peptides in the form of monomers [81] is not only non-toxic, but also has a positive effect on memory processes. The concentration of peptides increases as a result of intense brain activity and as a result of damage [82]. The degradation of AβT leads to the formation of peptides Aβ1-38 and Aβ1-40, which are hydrophilic [83], and Aβ1-42 and Aβ1-43, which are hydrophobic [84,85,86]. Under physiological conditions, the dominant peptide is Aβ1-40 with a low tendency to form fibrils and create amyloids [79]. The Aβ1-42 peptide is hydrophobic and considered an amyloidogenic form responsible for neurotoxicity [86]. The Aβ1-42 fragment self-associates into soluble oligomers that are toxic to neurons. The insoluble form of Aβ1-42 occurs inside the neuronal cell and undergoes conformational changes, transforming into Aβ plaques in Alzheimer’s patients’ brains.

Neurofibrillary tangles (NFTs) are aggregates of helical filaments (PHFs) of hyperphosphorylated tau protein, which play a role in axonal transport and microtubule stabilization [87,88]. In AD, tau aggregation leads to the breakdown of the microtubule network, axonal transport disorders [89], and the loss of neurons in the brain [90].

Inflammation of the nervous system, involving components of the immune system, such as microglia and astrocytes, also plays a role in the pathogenesis of AD. Activated microglia lose the ability to phagocytically remove Aβ protein aggregates [91,92,93] and release pro-inflammatory mediators, i.e., IL-1β: interleukin-1β, inducible nitric oxide synthase (iNOS), cyclooxygenase-2 (COX-2), IL-6: interleukin-6, and necrosis factor tumor alpha (TNF-α) [94,95,96,97]. LPS (lipopolysaccharide), which is a component of the cell wall of Gram-negative bacteria, is used to induce inflammation of the nervous system in in vivo studies on animal models [98]. LPS induces the formation of Aβ and the production of pro-inflammatory cytokines, which results in neuronal cell death and the appearance of cognitive disorders in mice [99,100]. Studies show that administering LPS to animals increases the production of ROS and Aβ by activating the NFκB signaling pathway [101,102].

The aggregation and hyperphosphorylation of tau protein is triggered by the activation of mitogen-activated protein kinase (MAPK) and NF-κB: nuclear factor kappa B, which are also mediated by microglial activation [103].

The impairment of memory and cognitive functions in AD is associated with synaptic dysfunctions and histopathological changes in the structure of dendrites, i.e., the loss of spines, abnormal sprouting, and dystrophic dendrites [104,105,106,107,108,109,110,111].

Genetic studies have selected four genes associated with AD, such as amyloid precursor protein (APP), presenilin 1 (PS1), presenilin 2 (PS2), and apolipoprotein E (ApoE). Presenilin PS1 is a hydrolytic component of γ-secretase [112] and mediates the intramembrane cleavage of APP [113], generating Aβ and senile plaques. The accumulation of Aβ under pathological conditions blocks ion channels and leads to mitochondrial oxidative stress. The disruption of energy metabolism ultimately causes neuronal cell death [114]. Mutations associated with these proteins lead to an increase in the production of Aβ peptides, which are responsible for, among others, the overfilling of neurons with calcium ions and neurodegeneration in AD. 

**Figure 1:**
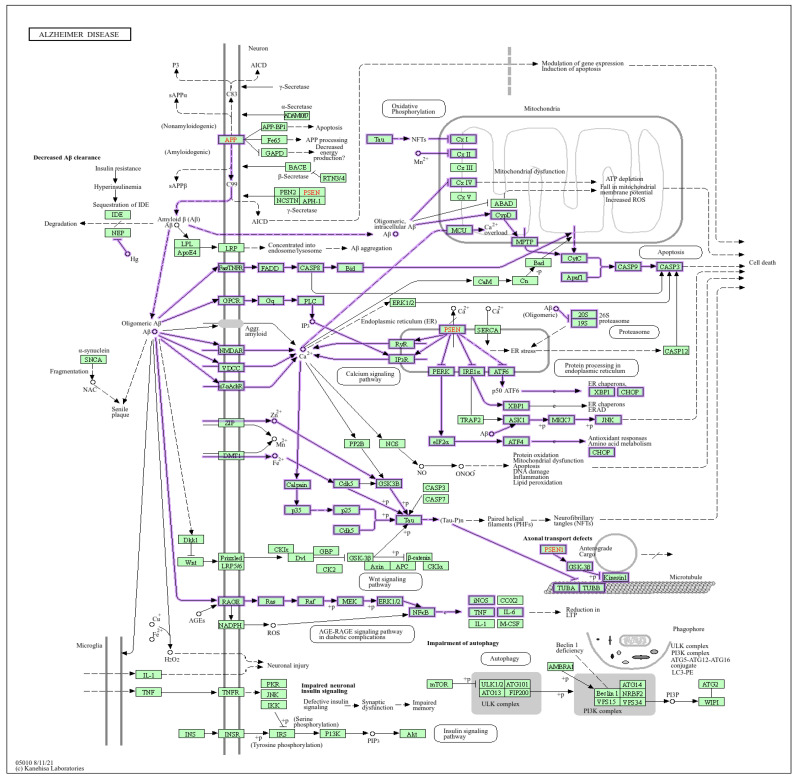
Alzheimer’s disease KEGG pathway (hsa05010; Alzheimer disease—Homo sapiens (human)) generated online at https://www.genome.jp/kegg-bin/show_pathway?hsa05010, accessed on 8 March 2024) [115].

The AD pathway generated using KEGG (Kyoto Encyclopedia of Genes and Genomes) [115] is presented in Figure 1.

## 3. AD Treatment Strategies

The treatment of AD involves the use of acetylcholinesterase (AChE) inhibitors such as galantamine, donepezil, rivastigmine, and the N-methyl-d-aspartate (NMDA) receptor antagonist, memantine [116,117].

Antibody therapies are currently in clinical trials, including humanized IgG1 antibody drugs like lecanemab, aducanumab, and donanemab. At the beginning of 2024, the U.S. Food and Drug Administration approved a donanemab (Kisunla) injection for the treatment of Alzheimer’s disease [118]. Aducanumab was approved by the FDA in 2021 to reduce Aβ plaques [119]. Two years later, in 2023, lecanemab was also approved for AD treatment. Other antibody drugs tested were withdrawn due to poor effectiveness and the risk of side effects, such as cerebral edema. Tacrine, a cholinesterase inhibitor, has been withdrawn in the US due to severe liver toxicity. According to ALZFORUM (www.alzforum.org, updated on 15 May 2024), eight AD therapies are currently in phase 4 clinical trials, 3 of which are dietary supplements.

There is currently a search for natural phytochemicals that could be effective in AD therapy and prevent neurodegeneration [120,121]. Substances with antioxidant, anti-inflammatory, and neuroprotective properties, characterized by easy bioavailability and the ability to cross the blood–brain barrier, are of great interest. Nutraceuticals, due to their anti-inflammatory and antioxidant effects, have been found to improve cognitive functions in humans by inhibiting neuronal apoptosis and the formation of senile plaques [122,123,124].

Lipopolysaccharide (LPS)-stimulated BV-2 microglial and N2a cells are activators of the NF-кB, MAPK, and IRF3 signaling pathways activating the expression of the pro-inflammatory gene through the release of cytoplasmic NF-кB.

Small molecules such as chrysin, 7,8-dihydroxyflavone (7,8-DHF), naringenin, luteolin, lycopene, ferulic acid (FA), ellagic acid (EA), caffeic acid (CA), gallic acid (GA), epigallocatechin-3-gallate (EGCG), theaflavins (TFs), and vanillin play a role in the pathways associated with Alzheimer’s disease (AD) [9]. They inhibit the activity of acetylcholinesterase (AChE) in the cholinergic pathway, which breaks down acetylcholine (ACh), a neurotransmitter that supports memory. These dietary components can promote the survival of neuronal cells by activating molecular signaling pathways, such as the mitogen-activated protein kinase (MAPK) pathways that control synaptic plasticity and neurogenesis, as well as by activating the Nrf2 (nuclear factor erythroid 2-related factor 2)-ARE (antioxidant response element) antioxidant system and the Akt (the phosphatidyl inositol 3-kinase (PI3K)/protein kinase B) signaling pathway, which is responsible for the balance between pro-apoptotic and anti-apoptotic proteins. Nutraceuticals also promote the autophagy of Aβ plaques and hyper-phosphorylated tau aggregates, as well as inhibit the secretion and expression of many inflammatory factors. Additionally, they are involved in the gut–brain axis through their interaction with neurotrophic and neurotransmitter systems. These small dietary molecules act against the apoptotic effect triggered by inflammatory cytokines, such as TNFα and IL-1β, or the TNFα/caspase-8/caspase-3 extrinsic pathways and cyto c/caspase-9/caspase-3 intrinsic pathways [9].

## 4. Carotenoids in AD Treatment

Carotenoids are natural compounds found in colorful vegetables and fruits, particularly those that are red, orange, yellow, and dark green, and in photosynthetic bacteria, some species of archaea, fungi, algae, and animals. Natural sources of common carotenoids and their contents are collected in Table 1.

There are over a thousand identified natural carotenoids, which can be categorized into carotenes with a hydrocarbon structure and xanthophylls, which have additional functional groups such as –OH, =CO, –CHO, –COOH, and others [143]. Carotenoids play a crucial role in protecting plants from damage caused by absorbing excess solar energy [144,145].

Carotenoids can absorb highly energetic blue/green light due to the presence of chromophore groups, expanding the absorption ability of plant pigments to the range of 330–1100 nm.

Humans obtain carotenoids from their diet. To improve the body’s absorption of carotenoids, it is important to properly prepare food in a way that breaks down cell walls, ideally by cooking it at a high temperature [146,147]. After being absorbed by the intestinal mucosa, they form complexes with proteins [148] or very-low-density lipoproteins (VLDLs) [149,150] and in this form, circulate throughout the body. That is why lutein, zeaxanthin, lycopene, α-carotene, β-carotene, and β-cryptoxanthin can be found in blood, brain tissue, or skin [51,151]. Lutein is the primary carotenoid present in the macula and brain. Carotenoids accumulate in the skin, providing protection against peroxide and free oxygen radicals (ROS) generated by exposure to ultraviolet (UV) radiation [152,153]. Numerous studies have shown that carotenoids protect against oxidative stress by inhibiting lipid peroxidation and DNA damage [154]. The photoprotective properties of carotenoids have been extensively studied both in vivo and in vitro [143]. Lycopene is believed to have the strongest photoprotective effect [153]. However, caution should be exercised when using carotenoid dietary supplements, as high levels of carotenoids can have a pro-oxidant effect by forming carotenoid radical cations (CAR^•+^), which can alter the structure and function of proteins [155]. Additionally, the excessive supplementation of beta-carotene has been associated with an increased risk of lung cancer [156].

There is evidence suggesting that carotenoids, due to their anti-inflammatory and antioxidant properties, may help prevent Alzheimer’s disease (AD) and cognitive decline. Long-term studies have shown that consuming carotenoids in the diet may protect memory and cognitive functions [157,158,159,160]. For instance, a 5-year prospective study involving 960 participants found a link between consuming lutein/zeaxanthin and β-carotene and a reduction in cognitive dysfunction [158]. Another study, part of the Memory and Aging Project (MAP) and initiated in 1997, examined the relationship between cognitive decline and long-term dietary carotenoid intake, including β-carotene, α-carotene, lutein-zeaxanthin, lycopene, and β-cryptoxanthin [161]. Participants obtained carotenoids from processed tomatoes, carrots, sweet potatoes, pumpkin, and dark green vegetables like kale and spinach. During the 2004–2018 observation period, 237 cases of AD were recorded. Participants with the highest carotenoid intake (24.8 mg/day) had a 48% lower probability of developing AD compared to those with the lowest intake (6.7 mg/day) (HR: 0.52; 95% CI: 0.33, 0.81; *p*-trend < 0.01). The study suggests a beneficial role of carotenoid intake, particularly lutein/zeaxanthin, in reducing the incidence of AD. Deceased participants who consumed more lutein/zeaxanthin and lycopene exhibited a lower level of AD neuropathology, such as amyloid plaque severity and neurofibrillary tangle density. Additionally, a higher carotenoid intake was found to be more effective in counteracting AD in men than in women (*p* = 0.02) [161].

Other long-term observations support the improvement of cognitive functions in AD with the consumption of lutein [157] and β-carotene [158]. Furthermore, supplementation with β-carotene, lutein, or lutein zeaxanthin has been shown to improve cognitive function in randomized, placebo-controlled trials [162,163].

The mechanism of neuroprotective effects of carotenoids is still under investigation. It is known that due to the conjugated double bond system, carotenoids possess antioxidant properties by stabilizing free radicals. Based on the molecular docking analysis of five carotenoids (cryptocapsin, lutein, fucoxanthin, astaxanthin, and bixin) to different forms of Aβ 42 and Aβ 40 fibrils, Lakey-Beitia et al. [38] revealed the existence of interactions between carotenoids and Aβ via hydrogen bonds and van der Waals interactions, through which carotenoids can inhibit the aggregation and disaggregation of Aβ. The proposed mechanism includes the blocking of Aβ peptide self-aggregation by the length (long polyene chain) and configuration of the compound (carotenoids in trans conformation, which increases their contact with Aβ peptide, oriented perpendicularly to Aβ peptide) and interactions of hydroxyl groups with Aβ peptide/fibril through hydrogen bonding and π–π interactions. The tested carotenoids interacted through van der Waals interactions with Ala21, Ile32, Gly33, Gly38, and Val39 residues and through the formation of hydrogen bonds with Glu22, Val24, Gly25, Asp23, Ser26, Lys28, and Leu34 residues of Aβ peptide. The binding strength, and consequently the ability to inhibit Aβ aggregation, depended on the configuration of hydroxyl groups and the presence of epoxy, allene, and ketone groups in the molecule. In the study, lutein was observed to be the most potent carotenoid against Aβ aggregation, due to its interactions with residues 20–28 of the Aβ peptide, which are essential for oligomerization. In each simulation, lutein showed the lowest CDOCKER energy, which is associated with a stronger interaction [38].

### 4.1. Carotens

#### 4.1.1. Lycopene

**Figure 2:**
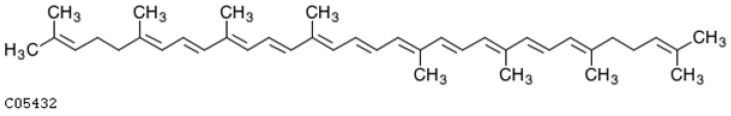
Chemical structure of lycopene–KEGG compound: C05432 [115].

Lycopene (Figure 2) is a red carotenoid that belongs to carotenes and is commonly found in fruits and vegetables. Tomatoes are a particularly rich source of lycopene. Lycopene exhibits neuroprotective, anti-inflammatory, and memory-improving antioxidant properties [164]. It has also been shown to have antihypertensive, cardioprotective, and anticancer effects. The recommended daily intake of lycopene is 5–10 mg [165]. Studies in animal models have not revealed any adverse effects from the ingestion of 3 g/kg per day [166,167]. Numerous reports suggest the potential use of lycopene in the treatment of AD [98,164,165,166,167,168,169,170,171,172,173,174,175,176,177,178,179,180] and other neurodegenerative diseases [181,182]. This is due to its antioxidant and anti-apoptotic activities [183,184], which help eliminate damage caused by Aβ [98,174] and enhance cognitive function [185,186].

From a chemical standpoint, lycopene is a lipophilic hydrocarbon with a high number of conjugated dienes [187]. Given its lipophilic nature, it is recommended to consume lycopene with fat to enhance its absorption [188]. Lycopene accumulates in various tissues in the human body, including the liver, lungs, prostate, colon, and skin, with its concentration being the highest compared to other carotenoids [189]. 

**Figure 3:**
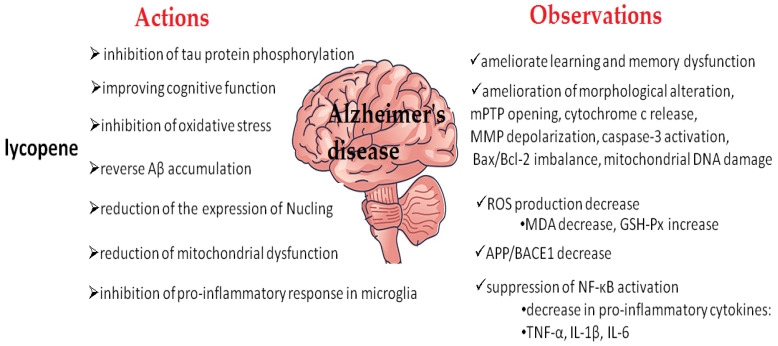
The neuroprotective effect of lycopene in Alzheimer’s disease (AD) models. Abbreviations: mitochondrial permeability transition pore (mPTP), reactive oxidative species (ROS), mitochondria membrane potential (MMP), nuclear factor-κB (NF-κB), and amyloid precursor protein (APP)/amyloid precursor protein lyase 1 (BACE1). Malondialdehyde (MDA) decreases and glutathione peroxidase (GSH-Px) increases. Antiapoptotic Bcl-2 (the B-cell lymphoma-2) protein family is involved in the control of intracellular Ca^2+^ signaling. A high level of Bax/Bcl-2 is associated with greater vulnerability to apoptotic activation.

According to a Norwegian study involving 261 participants across two age groups, the concentration of lycopene in plasma is about 0.78 µM [190]. Another study reports the concentration of lycopene in human serum to be approximately 0.5 μM [191]. In patients with AD, plasma lycopene levels, similar to other antioxidants (lutein, alpha-carotene, and beta-carotene), decreased compared to controls [192] in contrast to the increase in levels of 8-hydroxy-2’-deoxyguanosine (8-OHdG), a marker of oxidative damage to DNA, in peripheral lymphocytes [193]. These results indicate that AD patients have an increased state of oxidative stress with a low antioxidant status. Lycopene is capable of crossing the blood–brain barrier (BBB) into the central nervous system, making it a potential treatment option for neurological dysfunctions [194,195]. Many studies on the use of lycopene in neurodegenerative disorders have demonstrated its neuroprotective activity in the central nervous system (CNS) and improvements in cognitive performance [164,196,197,198,199]. Possible components of the neuroprotective effect of lycopene in AD are summarized in Figure 3.

Surprisingly, a systematic review and meta-analysis published in 2021 in *J. Alzheimers Dis.* [200] regarding the relationship between the level of carotenoids in plasma/serum and the occurrence of Alzheimer’s disease did not confirm significant differences in the level of lycopene between the study group and the control group (SMD = −0.12, 95% CI: −0.96 to 0.72, *p* = 0.78), even though the authors included 16 studies involving approximately 10,000 participants.

Improving the bioavailability of lycopene can be achieved by administering it orally in the form of lycopene micro-/nano-emulsion (LME). This form of the drug not only improves its bioavailability but also reduces its elimination rate [201]. In the study by Ning et al. [202] from 2021, the effectiveness of such a preparation was tested using a rat model of AD induced by Aβ. The study found an improvement in learning ability, an increased expression of proteins related to the Wnt/β-catenin pathway, and the promotion of neurogenesis in the DG and SVZ regions of the hippocampus.

The mechanisms responsible for the effects of lycopene are still being investigated mostly using animal models or cell lines. The neuroprotective effect is believed to be the result of restoring the activity of mitochondrial enzymes and eliminating oxidative stress, as lycopene is considered to be one of the most potent ROS scavengers [197,203]. 

The inhibition of oxidative stress and the alleviation of AD symptoms by lycopene has been confirmed in rodents [204,205]. Studies have been conducted on tau transgenic mice with the P301L mutation [196]. These P301L mice show AD-related memory deficits and an increase in MDA, a decrease in serum GSH-Px activity, and an increase in tau phosphorylation at Thr231/Ser235, Ser262, and Ser396 in the brain. 

Yu et al. [196] show that lycopene supplementation for 8 weeks (5 mg/kg) reversed these trends. Other authors also confirm the reduction in memory deficits, mitochondrial oxidative stress, and neuronal damage in Aβ1–42-exposed rats by lycopene [173,206,207]. Moreover, Hsiao et al. proved the effectiveness of lycopene in focal cerebral ischemia in rats [208]. 

The pro-inflammatory response in microglia induced by Aβ, which plays a role in the pathogenesis of AD, is also inhibited by lycopene. The neuroprotective effects of lycopene are mediated by the activation of microglia, the adenosine 5′-monophosphate-activated protein kinase (AMPK), peroxisome proliferator-activated receptor γ (PPARγ), and phosphatidylinositol-4,5-bisphosphate 3-kinase (PI3K)/protein kinase B (Akt) signaling [179,209]. Liu et al. [178] conducted a study in rats that showed that lycopene had a protective effect on β-amyloid-induced inflammatory responses in the choroid plexus. After intragastric lycopene supplementation for 21 days (5 mg/kg), cognitive functions improved in the study group and the cytokine profiles of tumor necrosis factor-α (TNF-α), interleukin-1β (IL-1β), and IL-6 were reduced in serum, and the inhibition of nuclear factor-κB (NF-κB) p65 and TLR4 activation in the choroid plexus occurred. The inhibition of the inflammatory response in the CNS and repair of learning and memory deficits as a result of 2 weeks of lycopene treatment was demonstrated in an in vivo study using rats exposed to Aβ 1-42 [176].

Lycopene supplementation (5 mg/kg, 21 days) reduced caspase-3 activation, which was induced by Aβ 1-42 injection in rats [175]. In another study by Wang et al. [98], approximately one month of treatment with lycopene at a ten times higher dose of 50 mg/kg prevented LPS-induced neuroinflammation and oxidative stress and reversed the accumulation of Aβ. Lycopene caused the increase in APP/BACE1 in the LPS-induced amyloidogenesis model in mice [98]. The effect of lycopene was also observed in vitro in cultured rat cerebral cortex neurons [173,174]. Lycopene was confirmed to reduce Aβ-induced neuronal damage by mitigating mitochondrial-related pathogenesis. Hwang et al. [170] proved that lycopene inhibited amyloid-β-induced changes in human neuroblastoma (SH-SY5Y) cells by reducing apoptotic responses in the form of an increase in the Bax/Bcl-2 ratio, caspase-3 activation, and p53 expression.

Fang et al. investigated the effect of lycopene on a cellular model of AD overexpressing Aβ [169]. The effect of lycopene was to activate the PI3K/Akt/Nrf2 signaling pathway and reduce BACE expression in M146L cells. Lim et al. [171] proved that lycopene inhibits apoptosis in calcineurin 1 (RCAN1)-overexpressing neuronal cells on chromosome 21 that are involved in the process of learning and memory [210,211]. The overexpression of the regulator of RCAN1 and oxidative stress cause neuronal impairment by a pro-apoptotic effect and may be a precursor of early AD [212,213]. 

One of the therapies for AD is the use of neural stem cells (NSCs) by secreting neurotrophic and immune-system-modulating factors and initiating cell turnover as precursors of neurons, astrocytes, and oligodendrocytes [214,215]. Huang et al. [172] showed that NSCs treated with lycopene secrete trophic factors (nerve growth factor (NGF), brain-derived neurotrophic factor (BDNF), and vascular endothelial growth factor (VEGF)) and reduce oxidative damage to neurons exposed to tert-butyl hydroperoxide (t-OHS). The anti-apoptotic effect was caused by the inhibition of the expression of Bax/Bcl-2, cytochrome C, and cleaved caspase-3.

#### 4.1.2. β-Carotene

**Figure 4:**
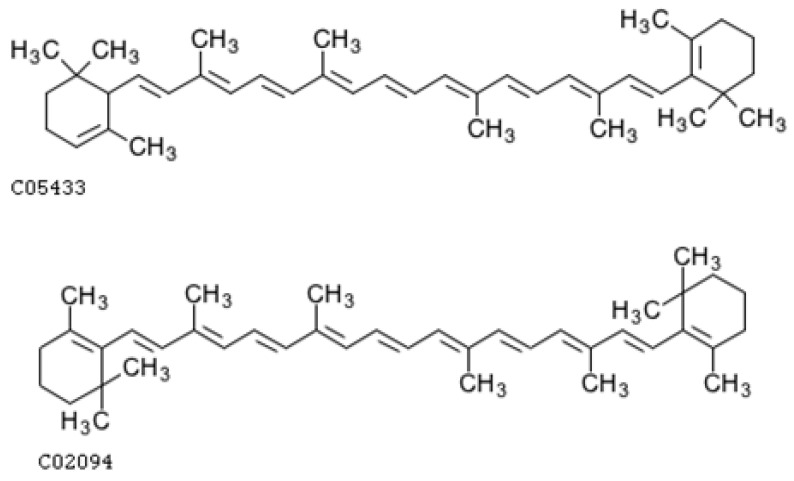
Chemical structures of α-carotene: KEGG Compound: C05433 (**top**), β-carotene-KEGG compound: C02094 (**bottom**) [115].

Alpha- (α-carotene) and beta-carotene (β-carotene) (Figure 4) are secondary metabolites synthesized by plants. These are carotene linear C40 hydrocarbons with a chain of conjugated double bonds. C-6′ in α-carotene between the e-end group and the conjugated double bonds is chiral, that is why all of α-carotene and its derivatives are (6′*R*) and (6′*S*) types. However, Shinichi Takaichi et al. [216], who examined the C-6′ chirality of naturally occurring α-carotene in 40 species using circular dichroism or nuclear magnetic resonance spectra, found they only had the (6′*R*) type.

α-carotene is abundant in carrot, orange, papaya, banana, and apricot, and β-carotene is found in carrot, lettuce, grapefruit, apricot, and broccoli [217]. β-carotene has an antioxidant effect by suppressing singlet oxygen and lipid peroxides [156,218]. In addition to β-carotene, its metabolites [219,220], i.e., active vitamin A compounds (ATRA), participate in changes in gene expression. ATRAs are formed as a result of the cleavage of β-carotene with the participation of β-carotene oxidase 1 (BCO1) at the central 15, 15” double bondand BCO2 at eccentric double bonds [221]. Retinoic acid (RA) interacts with nuclear receptors, e.g., retinoic acid receptor (RAR) and retinoid X receptor (RXR) [222]. Transcription factors such as NF-κB and nuclear erythrocyte factor 2-related factor 2 (Nrf2) also participate in β-carotene-mediated signaling [223]. Metabolites act as ligands for nuclear receptors. In this way, they are involved in the regulation of metabolic pathways, including lipidation, inflammation, and blood–brain barrier (BBB) integrity. Thanks to their influence on synaptic plasticity, they are involved in the regulation of cognitive function [224].

β-carotene is transformed into vitamin A, which is why it is often referred to as provitamin A. The metabolic conversion of β-carotene determines further retinoid-mediated signaling [223,225]. Vitamin A, apart from being involved in cell differentiation, regulating lipid metabolism and immune functions, is essential in the process of vision and regulating cognitive functions. The effects of β-carotene, as a precursor of vitamin A, on cognitive functions were summarized in a recent review by Wołoszynowska-Fraser in 2020 [226]. β-carotene is metabolized locally via retinol to retinoic acid in the hippocampus, an area where cognitive functions are impaired in early AD [227,228,229]. It has been shown in animal models that the role of retinoic acid in the hippocampus includes the induction of neurogenesis [230,231] and the correction of spatial memory deficits [232,233,234,235]. Moreover, vitamin A deficiency causes a reduction in hippocampal volume and the deposition of amyloid-β [236,237]. The biomarker used to assess the status of vitamin A in the body is the determination of retinol levels. Patients with AD not only have lower retinol concentrations [238], but also often lower concentrations of the retinol transport protein, RBP4, in their cerebrospinal fluid in the presence of inflammation [239,240,241].

Another active metabolite of vitamin A, retinoic acid (RA), is also a potent signaling molecule that plays a role in AD [242] by preventing neurodegeneration [243,244]. However, RA occurs in plasma at very low pmol/mL levels [245], in the form of several endogenous isomers, i.e., all-trans-RA, 9-cis-RA, 13-cis-RA, and 9,13-di-cis-RA; therefore, it is not often examined. Post-mortem studies have revealed retinoic acid receptor deficiency in people with AD [236]. Retinoic acid (RA)’ key isomers 9-cis RA (9CRA) and all-trans RA (ATRA) are ligands for the retinoic acid receptor (RAR). Additionally, 9CRA isomer is a ligand for another receptor, namely, retinoid X receptor (RXR) [246]. The synthetic RXR agonist bexarotene has been shown to improve the symptoms of Alzheimer’s disease [247,248,249]. 9-cis β-carotene (9CBC), which occurs mainly in the alga Dunaliella bardawil, is, like bexarotene, an RXR agonist, but unlike bexarotene, it is not cytotoxic [250,251]. The RXR heterodimer, together with another nuclear receptor RAR, is expressed in AD-prone regions mainly the basal forebrain and hippocampus, and their stimulation has several beneficial effects such as reducing neurodegeneration and promoting Aβ phagocytosis [252].

A study report on the effects of β-carotene on cognitive function and verbal memory, conducted by the Physicians’ Health Study, shows that the earlier age of β-carotene supplementation/or the longer duration of supplementation may provide cognitive benefits. The study, which lasted for 18 years, involved over 4000 participants over 56 years of age. The report also shows that annual β-carotene supplementation is not sufficient to improve global performance or verbal memory [160]. Similar results were found in the Nurse’s Health Study [238]. In case of low vitamin A intake, the use of supplementation improves cognitive function [253,254]. The concentration of β-carotene in the blood not only is positively correlated with telomere length and telomerase activity [255,256], but also reduces the risk of cognitive decline with age and the occurrence of Alzheimer’s disease [257]. 

A cross-sectional study [258] involving about two thousand participants reported improvements in cognitive function in a series of tests such as the Consortium to Establish Registry for Alzheimer’s disease (CERAD W-L), Animal Fluency Test (AFT), and Digit Symbol Substitution Test (DSST) after an α-carotene intake > 1379.5 mcg/d or a β-carotene intake > 7876 mcg/d). Also, a randomized controlled intervention known as the Mediterranean-DASH Intervention for Neurodegenerative Delay (MIND) (NCT02817074) confirmed that high plasma α-carotene concentrations are associated with better global cognition [259]. The study involved participants aged 65–84 years at risk of AD. The average plasma concentrations of α-carotene and β-carotene were 349.0 μg L^−1^ and 134.9 μg L^−1^, respectively. The study found that high plasma α-carotene levels were associated with a higher global cognitive score (*p* = 0.001). The global cognitive score improved successively as the plasma α-carotene concentration increased from 34.9 to 155.8 μg L^−1^ (*p* = 0.002). However, no statistically significant relationship was found between β-carotene and global cognition. 

Some authors postulate that the levels of α-carotene and β-carotene in the blood can be considered AD biomarkers, due to the fact that they are significantly reduced in AD [256,260,261,262]. Patients with reduced plasma β-carotene levels also show an increase in the levels of Aβ 1–42 and tau protein in their cerebrospinal fluid (CSF) [263]. However, the results presented by other authors on the levels of α-carotene, β-carotene, lycopene, and β-cryptoxanthin in people with AD compared to controls contradicted this; therefore, the use of carotenoids as AD biomarkers seems controversial [200,264,265]. The benefits of using β-carotene as a supplement in the prevention of AD are also controversial, as shown by a systematic review of the literature [266].

A case-cohort study by Koch et al. [265] did not confirm that plasma β-carotene and α-carotene were statistically significantly associated with AD, dementia risk, or cognitive decline. Surprisingly, trends toward a greater AD risk were observed with higher plasma levels of trans-β-carotene and α-carotene. Similar results were reported by Engelhart et al. [267] and the 2008 Nurses’ Health Study [268] regarding the association of plasma retinol with the incidence of AD. It should be noted that in the study by Hu et al. [257], the beneficial effect of β-carotene in improving cognitive functions was observed only among carriers of apolipoprotein E 4 alleles (APOE ε4).

In Wang et al.’s study [269], cognitive performance and reaction time are associated only with selenium levels (OR 1.047, 95% CI 1.005 to 1.091, *p* = 0.028) in contrast to other antioxidants, such as vitamin A, vitamin C, zinc, β-carotene, and urate.

The pathogenesis and therapeutic strategies of AD are also studied in animal models [270,271]. For this purpose, transgenic mouse models are used with mutations in the APP, PSEN1, or PSEN2 genes identified as the cause of early-onset AD, which affects at most 1% of patients [272,273,274]. Late sporadic AD, which occurs after the age of 60 years and is induced by various risk factors related to lifestyle and environmental pollution, is difficult to reproduce in animal models [275]. Many other factors, such as differences in the immune systems of mice and humans [276] and the reproduction of extensive neuronal loss in mouse models [277], make it difficult to transfer results obtained in animal models to humans. Nevertheless, studies using animal models have been carried out. An example is the report by Hir et al. [278], who used a mouse model of streptozotocin-induced AD, which was orally administered β-carotene at a dose of 1.02 and 2.05 mg/kg for 2 weeks. After this time, anti-oxidative effects, the inhibition of acetylcholinesterase, and a reduction of Aβ plaques were obtained. Another study is that of Twitto-Greenberg et al. [250] from 2024 using mouse models of AD, i.e., Tg2576, 5xFAD, and apoE4, which were treated with 9-cis beta-carotene (9CBC) in the form of powder from the algae Dunaliella bardawil. Thanks to such supplementation, improvements in long- and short-term memory, as well as reductions in Aβ plaques, tau hyperphosphorylation, and neuroinflammation were achieved.

The finding that higher serum β-carotene levels result in higher β-carotene-dependent signaling is partially disputed. There are other autoregulatory mechanisms in β-carotene/retinoid-dependent signaling [223]. Vitamin A status is thought to regulate the absorption and cleavage of β-carotene. If vitamin A intake is low, the uptake and conversion of β-carotene increases. In turn, hypervitaminosis A inhibits the conversion of carotenoids to retinal [279,280].

### 4.2. Xanthophyll Carotenoids

**Figure 5:**
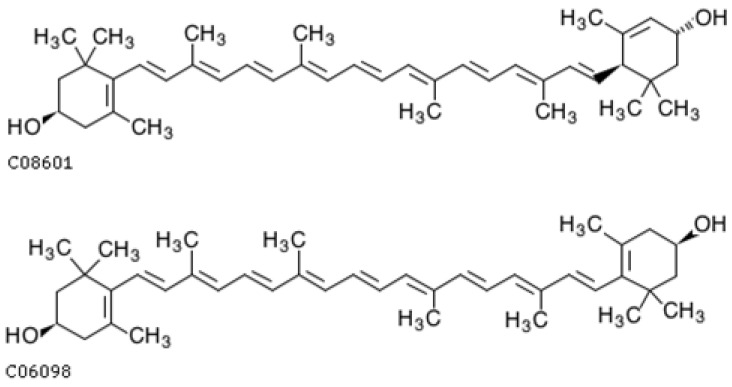
Chemical structures of lutein: KEGG compound: C08601 (**top**), and zeaxathin KEGG compound: C06098 (**bottom**) [115].

Lutein and zeaxanthin are xanthophyll carotenoid pigments that are structural isomers that differ in the position of one double bond (Figure 5). The position of the double bond in lutein causes the greater reactivity of the allylic hydroxyl end group compared to zeaxanthin, in which the double bond extends the system of conjugated double bonds. Thanks to the hydroxyl group, xanthophylls are more polar compared to carotenes, which affect ADME (absorption, distribution, metabolism, and excretion) processes and accumulation in tissues. Both lutein and zeaxanthin are strong scavengers of free oxygen radicals, especially singlet oxygen, which preferentially interacts with conjugated double bonds, thanks to the system of conjugated double bonds in its structure.

They are found in egg yolks and many fruits and vegetables that are dark green, e.g., spinach and lettuce. Lutein and zeaxanthin are found in the human body in the eye in the area of the macula and are responsible for the yellow color. They strongly absorb the highest-energy blue light, protecting the retina against damage. Their presence is essential; therefore, they must be supplied in one’s diet to prevent age-related macular degeneration [281,282,283]. However, in addition to affecting eye health, lutein may also be important for the brain, especially proper cognitive function. According to recent studies conducted on post-sectional brain tissues of elderly people over 70 years of age, the most abundant xanthophylls and carotene were β-cryptoxanthin and β-carotene [40]. However, people with AD had significantly lower concentrations of lutein (*p* = 0.03), zeaxanthin (*p* = 0.001), and anhydrolutein (*p* = 0.05). Healthy brains contained 1.5 and 2 times more lutein and zeoxanthin. The distribution of carotenoids in different areas of the brain is also not uniform. It turns out that the gray matter of the brain has statistically significantly higher concentrations of lutein and zeaxanthin compared to the white matter (*p* = 0.0006 and *p* = 0.009, respectively), and this relationship is also maintained in the course of AD. The authors of the study warn that zeaxanthin concentrations, next to lycopene, are the most deficient antioxidants in AD brains; therefore, the brain does not have sufficient antioxidant, anti-inflammatory, and anti-amyloidogenic protection [40].

Lutein/zeaxanthin deficiencies in the brains of people with AD are related to diet. As shown by the Memory and Aging Project [161], NHANESIII [264], EVA [284] studies, low intake is associated with low plasma levels and further deficiencies in these carotenoids in the brains of AD patients. People with higher levels of lutein/zeaxanthin have better results in cognitive tests [285,286,287], and the risk of AD is reduced by up to half [161,200]. There is evidence that lutein/zeaxanthin supplementation improves brain activity in older people [288,289]. Lutein/zeoxanthin deficiencies can be observed in the macular pigment [290]. While testing the level of carotenoids in plasma or the brain requires a multi-stage analytical procedure, taking whole blood from a vein, the content of lutein/zeoxanthin in the macula is assessed non-invasively based on the so-called “macular pigment optical density” (MPOD) using heterochromatic flicker photometry (cHFP) [290]. Studies on the relationship between MPOD and cognitive function were conducted in Ireland on a group of 4453 patients over 50 years of age [290]. The research confirmed that a lower MPOD was associated with, among others, a poorer prospective memory (*p* = 0.011) but not pictorial memory. Those with lower MPODs showed deteriorations in Montreal cognitive function (*p* = 0.016) and mental status (*p* = 0.026) [290].

#### 4.2.1. Lutein

Lutein, like other carotenoids, is transported after absorption in the digestive tract by lipoproteins [291,292]. Lutein has been detected in human brain tissue. Lutein-rich areas are those involved in cognitive functions including the cerebellum, brain bridge, and frontal and occipital cortical regions [293,294,295,296]. Lutein levels have been shown to be correlated with learning and memory functions [294]. Potential mechanisms for the neuroprotective effects of lutein have been proposed, highlighting the role of reducing oxidative stress and anti-inflammatory effects [296]. A congress in Lisbon in 2016 played a role in better understanding the biological role of lutein [297], at which specialists including Dr. Renzi-Hammond from the University of Georgia described AMD as “Alzheimer’s of the eye”. It should be noted that visual abnormalities such as AMD often coexist with AD and develop before cognitive decline occurs [298]. In turn, Professor Elizabeth Johnson, from the Jean Mayer USDA Human Nutrition Research Center on Aging at Tufts University in Boston, Massachusetts, emphasized that according to her research, “the brain preferentially takes up lutein compared to other carotenoids”.

A study by Leila Nazari et al. [299] from 2022 tested the preventive and therapeutic effect of lutein on cognitive disorders in a rat model of Alzheimer’s disease. The authors examined spatial memory (using the Morris water maze test (MWM) and Barnes test), passive avoidance memory and learning (using PAL tasks), and object recognition memory (using the novel object recognition (NOR) test). In addition to behavioral tests, measurements of the degree of lipid oxidation and serum antioxidant status were performed by determining the levels of malondialdehyde (MDA), total oxidative status (TOS), and total antioxidant capacity (TAC). The authors confirmed that lutein improves learning and memory and statistically significantly reverses the increase in MDA and TOS and the decrease in TAC observed in the AD group after the intravenous administration of Aβ [299]. Blood tests showed that MDA in the AD group was significantly higher (*p* < 0.01) compared to that in the control group. After the lutein treatment, a significant decrease in MDA was observed in the AD group (*p* < 0.05; F = 4.774) [299]. Also, Liu et al. confirmed the protective effect of lutein against Aβ(25–35) toxicity, probably by changing the expression of Nrf-2 and NF-κB in the endothelium of cerebrovascular cells [300]. There are many reports that lutein, in addition to zeoxanthin, has a positive effect on cognitive perception in older adults and people with AD [301], whose levels of these xanthophyll carotenoids are low [284].

Based on data from the National Health and Nutrition Examination Survey and the Centers for Medicare & Medicaid, Beydoun et al. [302] developed Cox proportional hazards regression models to examine the association between serum levels of vitamins A, C, E, and carotenoids and dementia in AD and other causes. Over 20 years of observations were conducted involving approximately 7000 people. The participants showed that only serum lutein and its structural isomer zeaxanthin levels were significantly associated with the risk of dementia (hazard ratio (HR) 0.93; 95% CI 0.87–0.99; *p* = 0.037). A similar relationship was shown between the level of β-cryptoxanthin in serum and dementia in two age groups (HR 0.86, 95% CI 0.80–0.93, *p* < 0.001 for 45+; HR 0.86, 95% CI 0.80–0.93, *p* = 0.001 for 65+) [302].

Over the last 10 years, the Pubmed database has included six clinical trials on the use of lutein in AD patients. A 2022, randomized, double-blind, placebo-controlled trial demonstrated behavioral and functional benefits following one-year daily supplementation with 1 g of fish oil (500 mg DHA, 150 mg EPA), 22 mg of carotenoids (10 mg lutein, 10 mg mesozeaxanthin, and 2 mg zeaxanthin), and 15 mg vitamin E [303,304]. The authors of the study emphasize that the study group recorded a decrease in the severity of AD. Statistical significance between the groups was achieved for clinical memory parameters (*p* < 0.001).

Xanthophyll carotenoid supplementation is particularly effective in AD therapy when administered together with omega-3 fatty acids. The study by Nolan et al. [305] tested 12 AD patients who were supplemented with xanthophylls for 1.5 years (lutein:meso-zeaxanthin:zeaxanthin 10:10:2 mg/day) and 13 AD patients who received supplementation with xanthophyll carotenoids and fish oil (lutein: meso-zeaxanthin:zeaxanthin 10:10:2 mg/day plus 1 g of fish oil (430 mg docohexaenoic acid (DHA) and 90 mg eicopentaenoic acid (EPA))). In the second group, not only was a higher level of xanthophyll achieved (*p* < 0.05), but the progression of AD was slowed (*p* = 0.003) in terms of memory, vision, and mood. It is interesting that the same group of researchers in an earlier study from 2015 [295] did not find significant changes in cognitive function (*p* > 0.05) in AD patients supplemented with macular carotenoids at an identical dose of 10 mg meso-zeaxanthin + 10 mg lutein + 2 mg zeaxanthin. However, it seems that the key factor in this case is the duration of supplementation, which, in the last study, was much shorter and amounted to only 0.5 year. This period of supplementation was only sufficient to improve serum xanthophyll concentrations (*p* < 0.001), as well as visual acuity and contrast sensitivity (*p* = 0.039) [295].

Similarly, no statistically significant effect of supplementation with long-chain polyunsaturated fatty acids (LCPUFAs) (1 g) and/or lutein (10 mg)/zeaxanthin (2 mg) on cognitive function was reported in another double-blind, randomized clinical trial [306].

Despite these inconsistencies, the Mediterranean-DASH Intervention for Neurodegenerative Delay (MIND) study clearly demonstrated that high levels of both plasma α-carotene and lutein and zeaxanthin were positively associated with higher semantic memory scores (the *p* value for this trend was 0.009) [259].

Lutein is considered the most anti-amyloidogenic carotenoid that inhibits the formation of Aβ 42 fibrils. Katayama et al. believe that it is the number and configuration of hydroxyl groups that determine its power to inhibit Aβ aggregation [307]. Therefore, lutein with two hydroxyl groups has a higher activity than, for example, β-cryptoxanthin with one hydroxyl group, carotenes such as β-carotene and α-carotene, or apocarotenoid (bixin) with a *cis* configuration. Studies based on molecular docking simulations [38] found that lutein interacts with Aβ through hydrogen bonds and van der Waals interactions, providing the inhibition of Aβ aggregation and amino acids which are necessary for oligomerization. The long polyene chain of lutein in the *trans* configuration and perpendicular orientation, which is longer than the Aβ peptide, and two hydroxyl groups interacting with the polar residues of the Aβ peptide block the self-aggregation of the peptide.

Due to its antioxidant and anti-inflammatory properties, lutein has the potential to protect against AD. An obstacle to its widespread use is its poor solubility, which causes low bioavailability [308]. Dhas and Mehta [309] developed core/shell nanoparticles (Chitosan@PLGA C/SNPs) containing lutein, chitosan and biodegradable polymers, and poly (lactic-co-glycolic acid) (PLGA) for intranasal administration. In vitro studies showed that the sustained-release preparation penetrates the BBB and can be efficiently delivered by endocytosis.

#### 4.2.2. Astaxanthin

**Figure 6:**
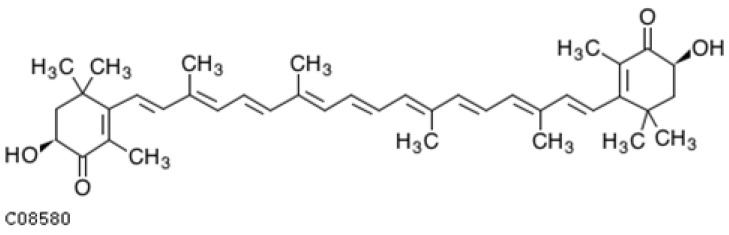
Chemical structures of astaxanthin–KEGG compound: C08580 [115].

Astaxanthin (Figure 6) is a xanthophyll carotenoid with antioxidative and anti-inflammatory effects, occurring naturally in seafood and marine algae such as the algae Haematococcus pluvialis. Astaxanthin is absorbed in the small intestine and reaches the brain by crossing the BBB [310]. Astaxanthin uses all lipoprotein densities for transport [311]. The effect of astaxanthin on brain function has been analyzed in humans and animal models, mainly rodents [312,313].

Various doses of astaxanthin have been tested in clinical trials, ranging from 2 mg to 12 mg daily (Table 2). Studies have tested various properties of astaxanthin to lower LDL cholesterol levels and to help fight atherosclerosis, hypertension, cancer, and diabetes [120,314,315,316,317]. 

Clinical and preclinical studies conducted in humans mostly confirm that astaxanthin prevents dementia and cognitive aging [120]. However, although improvements are observed on various measurement scales, such as the ADAS-Cog scale, the studies either lack a control sample [318], observe only slight improvements in memory, reaction time, and delayed recall [319], or involve too few study groups [320]. In turn, in the study by Katagiri et al. [320], memory improvements are visible compared to the baseline but not to the control group.

There are also studies [231] reporting no significant improvement in cognitive functions compared to a placebo group [321,322] or reporting improvements observed in memory tests in people under 55 years of age treated with astaxanthin as opposed to older people [322]. 

Another study [323] was conducted on healthy elderly people. The authors measured the level of phospholipid hydroperoxides (PLOOH) in erythrocytes, a specific marker of oxidative damage to the cell membrane. The results confirmed that astaxanthin supplementation reduced PLOOH levels. The same group of researchers in a subsequent study [324] hypothesized that β-amyloid peptide (Aβ) impairs the ability of red blood cells (RBCs) to transport oxygen to the brain and examined the levels of Aβ40 and Aβ42 in red blood cells and blood plasma. The Aβ concentration in RBCs determined by an ELISA was 5.32 ± 0.21 pmol/g hemoglobin and 2.09 ± 0.06 pmol/g hemoglobin for Aβ40 and Aβ42, respectively, and increased with age. Aβ levels in red blood cells were approximately eight and fourteen times higher than in plasma for Aβ40 and Aβ42, respectively. It was shown that after supplementation with astaxanthin at a dose of 12 mg/day for 12 weeks, there was a reduction in Aβ concentration in red blood cells. Unfortunately, astaxanthin supplementation had no effect on plasma Aβ40 and Aβ42 levels [324].

The effectiveness of astaxanthin against dementia or MCI is controversial [325]. The significant improvement in cognitive function observed in many studies thanks to astaxanthin supplementation is the driving force for further attempts. Few studies concern combined supplementation with an additional ingredient, e.g., sesamine, which, among other things, protects astaxanthin against immediate degradation [321]. Bhatt et al. prepared astaxanthin solid lipid nanoparticles, which showed strong neuroprotective effects in neuronal cell lines after intranasal administration [326].

The beneficial effect of astaxanthin on cognitive deficits and memory improvements was also tested in animal models. Zhang et al. [327] administered different doses of Haematococcus pluvialis algae powder (0, 0.26, 1.3, and 6.4 mg/kg body weight) to BALB/c mice for 30 days. The mice were tested in the Morris water maze. Experimental results show that a preparation containing astaxanthin can help improve memory in a dose-dependent manner.

Komaki et al. [328] checked how selected antioxidants, i.e., vitamins C and E, and 600 mg/kg astaxanthin can eliminate the adverse effects of a high-fat diet (HFD), which induces oxidative stress. Wistar rats were tested for passive avoidance learning (PAL) using a shuttle apparatus. After half a year of following the diet, the results revealed that the supplementation effectively prevented the PAL impairment that was caused by the HFD.

The authors of the research indicate that astaxanthin is responsible for anti-inflammatory [329,330,331] and antioxidant effects [332,333,334,335,336]. The anti-inflammatory effect of astaxanthin is due to its ability to inhibit the nuclear translocation of NF-κB and to reduce the expression of TNF-α in the hippocampus and frontal cortex [330]. Astaxanthin protects against oxidative damage by modulating the intracellular Keap1-Nrf2 pathway and activating the nuclear transcription factor of erythroid type 2-Nrf2, which is one of the key elements of the cell’s response to oxidative stress [336].

The neuroprotective and memory-enhancing effects of astaxanthin were confirmed in a rat model induced by the potent neurotoxin doxorubicin (DOX) [337]. Astaxanthin at a dose of 25 mg/kg showed a protective effect against induced cognitive disorders. An improvement in the structure of the hippocampus, a reduction in acetylcholinesterase activity, and a weakening of neuronal apoptosis were observed [337].

Studies in mouse models of traumatic brain injury (TBI), as well as cerebral ischemia/strokes and subarachnoid hemorrhage (SAH) are promising [338]. The oral administration of astaxanthin at a dose of 25 or 75 mg/kg for a month resulted in improvements in, among others, cognitive function (measured using the object recognition test and the Y-maze test) [338]. Astaxanthin reduced neuronal loss and affected the levels of brain-derived neurotrophic factor (BDNF), growth-associated protein-43 (GAP-43), synapsin, and synaptophysin (SYP) [338]. Also, in the case of cerebral ischemia/reperfusion, astaxanthin at a dose of 10 mg/kg/day was effective in eliminating memory deficits [339], thanks to the improvement in antioxidant potential. The attenuation of oxidative stress by astaxanthin is demonstrated by reduced malondialdehyde levels, an increase in the levels of the endogenous antioxidants, reduced glutathione and superoxide dismutase in the hippocampus, a decrease in the expression of cytochrome C (Cyt C), cleaved caspase-3, and Bax, and an increase in the expression of Bcl-2 [339].

Wen et al. [340] were the first to provide evidence that astaxanthin may be helpful in the treatment of AD because it protects against glutamate-induced cytotoxicity. The neuroprotective effects of astaxanthin were mediated by Akt/glycogen synthase kinase-3β (GSK-3β) signaling in HT22 cells of the HT-22 mouse hippocampal neuronal cell line, attenuating caspase activation and mitochondrial dysfunction [340].

#### 4.2.3. Fucoxanthin

**Figure 7:**
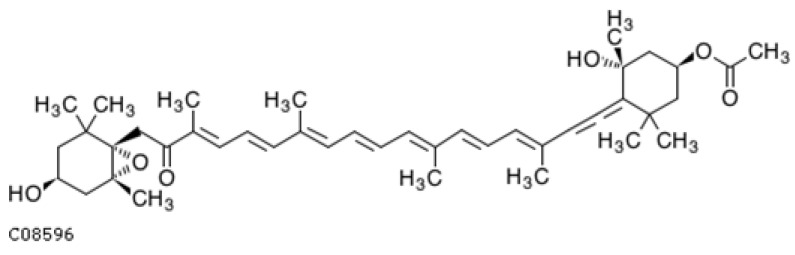
Chemical structure of fucoxanthin–KEGG compound: C08596 [115].

Fucoxanthin (Figure 7) occurs in the chloroplasts of brown algae [341]. Reports show that fucoxanthin has neuroprotective effects in AD [42,342,343,344]. Unfortunately, the first preclinical and clinical studies confirmed low toxicity but also low bioavailability in the CNS. Fucoxanthin in the form of nanoparticles overcomes this problem. The intravenous injection of nanoparticles improves cognitive functions in AD mice and inhibits the formation of amyloid-β fibrils and oligomers [345].

Although fucoxanthin’s strong antioxidant and anti-inflammatory properties have been confirmed, it is a relatively poorly researched carotenoid. The antioxidant activity of fucoxanthin and its metabolites, especially against hydroxyl radicals, is many times stronger than that of α-tocopherol [346]. Metabolic studies are mainly performed in in vivo mouse and rat models [347,348,349]. The BBB penetration and effectiveness of fucoxanthin in reversing brain damage were confirmed in a study in animals with middle cerebral artery occlusion (MCAO) [350]. It has been noted that in cases of neurodegeneration and ischemia, fucoxanthin restores cognitive and motor functions [178,191,204].

Several studies have confirmed the anti-apoptotic effect of fucoxanthin [351] as a result of an increase in Bcl-2 abundance and a reduction in Bax activity, cytochrome c, and caspase 3 release [350,352]. Fucoxanthin provides neuroprotection by activating the Nrf2 transcription factor and the PI3K/Akt pathway involved in the regulation of antioxidant genes [353,354].

Studies based on computational structure and molecular docking simulations revealed that fucoxanthin binds through hydrogen bonds and van der Waals interactions with residues 16–20, 24–28, and 33–37 to the Aβ peptide and inhibits aggregation and the formation of pathological Aβ fibrils [38]. However, this activity is lower compared to lutein, bixin, cryptocapsin, and astaxanthin, mainly because fucoxanthin possesses hydroxyl groups in other positions, but also because of other groups that could influence activity, such as an epoxide group or an allene group [38].

#### 4.2.4. β-Cryptoxanthin

**Figure 8:**
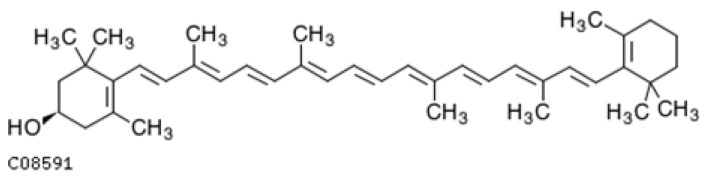
Chemical structure of β-cryptoxanthin–KEGG compound: C08591 [115].

β-Cryptoxanthin (Figure 8) belongs to the xanthophyll group with one hydroxyl group molecule and 11 conjugated double bonds. It can be considered as a monohydroxy derivative of β-carotene, albeit more polar. Similarly to other carotenoids, it occurs in yellow-orange fruits and vegetables as free β-cryptoxanthin and in an extrified form. The main sources of β-cryptoxanthin are pumpkin, persimmon, papaya, pepper, mango, apricot, mandarin, and orange [355,356]. However, it also occurs in green leafy vegetables (cilantro, parsley, and basil), and as well as in animal products including chicken skin, egg yolks, and butter [357,358].

In recent years, there has been a growing interest in β-cryptoxanthin due to its good bioavailability, which is higher than that of other carotenoids [359]. Similar to beta-carotene, β-cryptoxanthin is a precursor of vitamin A and is metabolized in the body to retinol [360].

Studies have shown that there is an inverse relationship between β-cryptoxanthin and the risk of many lifestyle diseases, such as cancer [361], diabetes [362], liver diseases [363], and cognitive function [364]. It should be emphasized that the pure form of β-cryptoxanthin is unstable and degrades under the influence of light, heat, and oxygen [360].

In the human body, β-cryptoxanthin occurs in human blood and tissues, and the ester form is enzymatically hydrolyzed in the intestines with the participation of pancreatic lipases [365,366]. The level of β-cryptoxanthin in the blood is not constant and depends on one’s diet. Higher levels of β-cryptoxanthin were observed in Japanese people, which is related to the consumption of satsuma mandarins rich in β-cryptoxanthin [367], approximately 1.8 mg/100 g [368].

Thanks to its chemical structure characterized by a system of conjugated double bonds, βcryptoxanthin has antioxidant properties and is more effective than vitamin E in quenching singlet oxygen [369]. Studies confirm the activity of βcryptoxanthin in reducing the effects of oxidative stress and lipid peroxidation [370].

**Figure 9:**
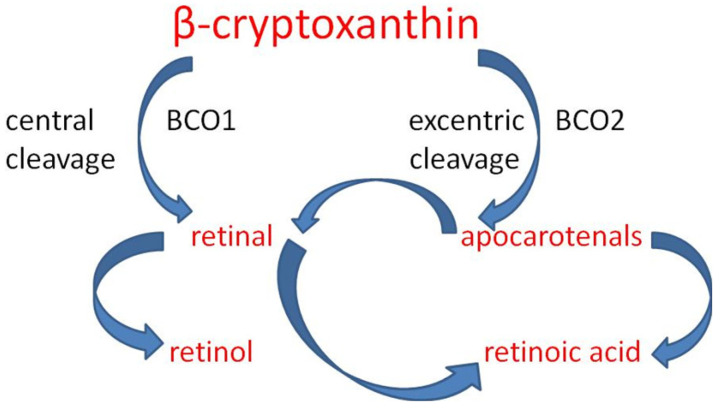
The cleavage pathways of β-cryptoxanthin.

α-,β-cryptoxanthin is a source of vitamin A due to the fact that it has an unsubstituted β ring, similar to α-, β-, and γ-carotene. In the body, it is enzymatically cleaved with the help of (BCO1) and (BCO2) to retinol, retinal, and retinoic acid, which is a key factor in neuronal differentiation [371,372] (Figure 9). Unlike β-carotene, which splits into two retinol molecules, β-cryptoxanthin forms only one [369], but due to its better bioavailability, it is considered a rich source of vitamin A [373].

May A. Beydoun et al. [302] used data from studies conducted by the National Health and Nutrition Examination Surveys, Medicare, and Medicaid to test the hypothesis whether there is a relationship between the presence of various antioxidants in blood serum, i.e., vitamins A, C, and E and carotenoids, and incidents of AD and dementia among adult patients in the USA. The study covered a period of 17 years, with over 7000 patients aged 45–90. The study showed an inverse relationship between the level of β-cryptoxanthin in serum and dementia regardless of age and gender (HR 0.86, 95% CI 0.80–0.93, *p* < 0.001 for 45+; HR 0.86, 95% CI 0.80–0.93, *p* = 0.001 for 65+). In addition to β-cryptoxanthin, similar conclusions were found for other carotenoids, such as luteins + zeaxanthins. A 2021 study [161] confirmed the existence of such a relationship, but for β-cryptoxanthin, it was only marginally significant. This study focused on postmortem brain AD neuropathology and included just over 500 cases.

In a systematic review and meta-analysis by Lin Wang et al. [374], the hypothesis was tested whether low levels of carotenoids in the blood correlate with dementia and mild cognitive impairment. The study authors searched electronic databases such as the Web of Science, PubMed, Embase, and the Cochrane Library and collected data published through early 2023. Ultimately, 23 studies were included in the analysis (*n* = 6610). Results from 1422 patients with dementia, 435 patients with MCI, and 4753 controls were included. Based on a meta-analysis, they showed that patients with dementia have reduced levels of carotenoids in the blood compared to controls. In addition to lycopene, α-carotene, β-carotene, lutein, and zeaxanthin, the level of β-cryptoxanthin (SMD: −0.617; 95% CI: −0.953, −0.281) was significantly negatively correlated with the examined dementia indicators [374].

Human brains contain several carotenoids, most of them from the xanthophyll group. The highest concentrations are β-cryptoxanthin, lutein, anhydrolutein, and zeaxanthin [375]. The study by Dorey et al. [40] shows that there is no statistically significant difference between the level of β-cryptoxanthin not only between the gray matter and white matter of the brain, but also between healthy people and those suffering from AD. However, the level of retinol, which is a metabolite of both β-cryptoxanthin and β-carotene, is statistically significantly (*p* = 0.006) lower in those with AD compared to controls. Retinol deficiency in the gray matter of the AD brains was significant, as retinol in the AD brains represented only 46.5% of the content of healthy brains (*p* = 0.003). The authors of the study detected a very high level of an unidentified chemical compound with the spectral characteristics of xanthophyll, which was significantly higher in the AD brains than in the controls (XMiAD; *p* = 0.006) but only in the gray matter of the brain [40].

#### 4.2.5. Crocin and Crocetin

**Figure 10:**
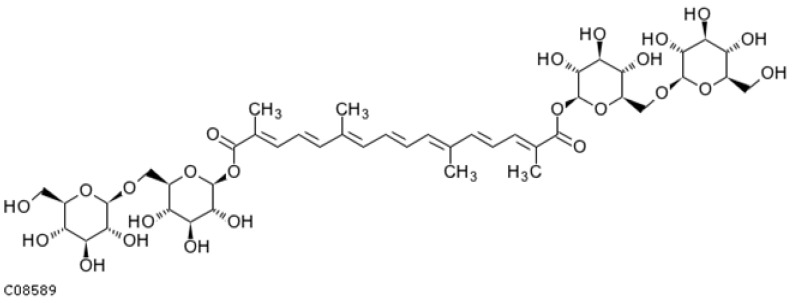
Chemical structure of crocin–KEGG compound: C08589 [115].

Crocetin and crocin (digentiobiosyl ester of crocetin) (Figure 10) are carotenoid pigments of saffron (*Crocus sativus* Linne) and gardenia fruit (*Gardenia jasminoides* Ellis). Apart from the conjugated polyene chain, as in crocetin, crocin is distinguished by the presence of sugar substitutes, which makes it soluble in water. A bright red crocin gives the stigmatic lobes of saffron crocus flowers their red color [376]. Many previously published studies have shown that safflower oil has antioxidant properties and supports learning and memory [377]. Both compounds have been tested in medication for many neurological disorders [378,379,380,381].

When testing extracts, it should be taken into account that saffron, in addition to crocetin and crocin, also contains conventional carotenoids such as α and β-carotene, lycopene, and zeaxanthin, and other ingredients such as sugars, proteins, amino acids, fats, minerals, essential oil (safranal), and bitter substances (picrocrocin) [382].

The activity of saffron extract was tested by in vitro enzymatic and molecular docking [383]. Moderate acetylcholinesterase (AChE) inhibitory activity was observed (up to 30%). However, in silico docking studies showed that safranal interacts only with the AChE binding site, and crocetin and dimethylcrocetin bind to the catalytic sites and peripheral anions. IC(50) values were also determined, which were 96.33, 107.1, and 21.09 μM for crocetin, dimethylcrocetin, and safranal, respectively. Other in vitro studies have shown that crocin has an affinity for the N-methyl-D-aspartate (NMDA) ion channel receptor in rat hippocampal neurons [384]. The antagonist of this NMDA receptor is, among others, memantine, a drug used to treat cognitive deficits in AD. Crocin acts against synaptic dysfunction by antagonizing NMDA receptors’ depression [384,385].

In the study by Nikolaos Pitsikas et al. [386], extracts of *Crocus sativus* L. at a dose of 30 mg/kg and 60 mg/kg were shown to be able to antagonize memory deficits induced by an amnesic (scopolamine). In the previous study, higher doses of the extract (125–500 mg/kg) were also tested, which were effective in the object recognition test and ethanol-induced performance deficits [387]. In a streptozocin (STZ)-induced sporadic AD model, the administration of crocin at a dose of 30 mg/kg antagonized AD in rats [388]. In the study by Hosseinzadeh and Ziaei [389], the effect of an aqueous extract of *Crocus sativus* stigma (0.0025–0.56 g/kg), safranal (0.2–0.75 mL/kg), and crocin (50 and 200 mg/kg) on scopolamine-induced learning deficits was assessed. According to the authors of the study, even a much smaller dose of 5 mg/kg of the extract shortened the latency time in rats performing the Morris water maze task. In turn, hyoscine-induced memory deficits (1 and 500 mg/kg of hyoscine) were reduced by the stigma extract and crocin at both low and high doses. However, all the preparations tested had no effect on intact memory.

Chronic stress causes functional and structural changes in the limbic system, which is responsible for memory and cognitive functions. Recent studies have shown a link between the development of AD and chronic stress. Experimental studies confirm the effectiveness of saffron/crocin in the fight against the decline in cognitive function caused by stress. The mechanisms responsible for this activity include the following, among others: antioxidant and anti-inflammatory effects, the inhibition of acetylcholinesterase, the aggregation of Aβ and tau proteins, and the promotion synaptic plasticity [390].

Crocin improves learning and memory and prevents hippocampal damage resulting from chronic stress in rats [391,392] by increasing GPx and SOD activity and reducing plasma corticosterone levels [393,394]. The antioxidant activity of crocin is responsible for its effect. By scavenging free radicals, reducing the production of peroxides membrane lipids, and restoring SOD activity, crocin prevent cerebral ischemia and damage to the cerebral cortex and hippocampus [395]. Crocin is also responsible for anti-inflammatory effects by suppressing microglial activation and increasing CX3CR1 expression by suppressing NF-κB initiation [396]. Asadi et al. [397] investigated the effect of crocin on memory, cell apoptosis, and autophagy by the use of in vivo models of AD. The results confirm that crocin improves spatial memory indicators and inhibits apoptosis induced by beta-amyloid, as evidenced by a reduced Bax/Bcl-2 ratio and the level of cleaved caspase-3 [397]. However, autophagy biomarkers Beclin-1 and the LC3-II/LC3-I ratio do not change under crocin administration [397]. An in vivo study in rats by Rezai et al. [398] showed that the administration of 25 mg/kg of crocin can inhibit the effect of morphine, which reduces the expression of BDNF and CREB genes in the ventral tegmental area (VTA).

Crocetin is a dicarboxylic acid, the aglycone of crocin. Crocetin has seven conjugated double bonds and the possibility of isomerization into the cis form and the trans form, which is more stable. It also has proven neuroprotective effects in AD in in vitro, in vivo, and human studies [399]. 

In 2021, Wani et al. [400] conducted comprehensive studies that confirmed the effect of crocetin as an autophagy inducer in AD mediated by the STK11-AMPK activation pathway. The activation of autophagy allowed for the removal of aggregated and hyperphosphorylated proteins. The studies were performed on different types of brain cells (N9 microglial and primary neuron cells) in the brains of wild-type mice (male C57BL/6 mice) and confirmed in a mouse model of AD (5XFAD transgenic males). A 30-day treatment with crocetin at three different doses (5, 10, and 20 mg/kg) was confirmed to reduce Aβ levels and neuroinflammation. Additionally, an improvement in memory function was observed under the influence of the crocetin treatment. 

Zhang et al. [401] investigated the effects of crocetin on inflammation and amyloid-β (Aβ) accumulation. The study was performed in a mouse model of AD in transgenic APPsw mice. Mice with the Swedish mutant of the APP751 transgene were treated orally with various doses of crocetin 0–30 mg/kg/day for half a year. Many positive effects of treatment were observed in the form of improved memory and learning, which resulted in reduced Aβ, lower levels of pro-inflammatory cytokines, and the inhibition of NF-κB activation and P53 expression in the hippocampus. Studies dedicated to the anti-AD effects of products coming from saffron divided into experiments performed with humans, animals, and cell lines are collected in Table 3.

The use of crocetin in pharmaceutical preparations is limited by its low stability and poor solubility. Liu et al. [416] compiled special inclusion complexes with three cyclodextrins (α-CD, HP-β-CD, and γ-CD) to overcome these problems. In this way, they achieved a three to four times increase in bioavailability in studies on rats. Bharate et al. [417] prepared a preparation from Crocus sativus extract in the form of gelatin capsules, which, by inducing P-gp expression, increased the clearance of amyloid-β in AD brains. Song et al. [418] prepared crocin-loaded nanoparticles that reduced amyloid-β concentration in differentiated SH-SY5Y cells. Wong et al. [419] synthesized a crocetin-γ-cyclodextrin inclusion complex, which, when injected intravenously, crossed the BBB and reduced the production of amyloid-β protein.

### 4.3. Carotenoid-Based Treatment

Carotenoids, like other phytochemicals, are being investigated as potential drugs. An overview of registered clinical trials on carotenoids is summarized in review articles [119,420,421]. The authors found 193 studies on carotenoids on ClinicalTrials.gov and ISRCTN. Most of the research concerned food, mainly tomatoes and tomato products. Currently, the most popular topics are lutein, lycopene, and astaxanthin. The tested pharmaceutical formulations are proposed for the treatment of eye and circulatory system health. Newer trends include applications to improve cognitive functions and intestinal microbiota.

Currently, medicines and supplements containing carotenoids include preparations with lycopene dedicated to skin diseases (IQQU SPF 50 sunscreen) and a dietary supplement intended for patients with hypertriglyceridemia. There are many preparations containing a derivative of vitamin A, e.g., isotretinoin (Absorica, Accutane, Amnesteem, Claravis, Clarus, Epuris, Myorisan, Sotret, and Zenatane), acitretin (Soriatane), etretinate (Tegison), and tretinoin (Altreno, Atralin, Avita, and Renova), intended for the treatment of acne and psoriasis. In the treatment of eye diseases, there are preparations with carotenoids, such as Vitamin A palmitate, intended for the prevention of cataracts, and retinitis pigmentosa and zeaxanthin (AREDS2 Vista), intended for the treatment of age-related macular degeneration (AMD)/cataracts, glaucoma, non-proliferative diabetic retinopathy/diabetes type 2. A preparation with β-carotene (Pregvit) is used for adjunctive treatment of photosensitivity in patients with erythropoietic protoporphyria.

Although there is evidence of the effectiveness of carotenoids in improving cognitive functions, there is no AD therapy with their participation, only nutritional recommendations [422,423]. The reason may be the fact that the research presented so far is correlational and does not indicate a cause-and-effect relationship.

A new direction is certainly given by the recent discovery by Tijms’s research group [424], which identified five different biological variants of AD that respond differently to treatment. Understanding the heterogeneity of AD at the molecular level is crucial for developing effective therapy. Subtypes were distinguished by mass spectrometry proteomics in cerebrospinal fluid. The subtypes differ in the increase/decrease in amyloid production, impaired BBB permeability, RNA dysregulation, and choroid plexus dysfunction. 

The problem is to determine reference ranges for carotenoids in blood plasma, which would be helpful in interpreting the obtained results.

Burrows et al. [425] in 2015 published a systematic review of studies on carotenoid levels in plasma based on 142 cross-sectional, cohort, case-control, and randomized controlled trials (RCT) studies involving 95,480 participants. The authors of the study did not observe a relationship between the intake of carotenoids and their concentration in plasma. However, the results of the meta-analysis are valuable because they present weighted average carotenoid concentrations in plasma, which were as follows: lycopene 0.62 μmol/L (95% CI: 0.61, 0.63, *n* = 56 studies), β-carotene 0.47 μmol/L (95% CI: 0.46, 0.48, *n* = 78), lutein/zeaxanthin 0.31 μmol/L (95% CI: 0.30, 0.32, *n* = 31), cryptoxanthin 0.17 μmol/L (95% CI: 0.17, 0.18, *n* = 44), and α-carotene 0.12 μmol/L (95% CI: 0.11, 0.13, *n* = 53).

Mullan et al. [426] performed a meta-analysis of 52 studies using random-effects models of cumulative mean differences in antioxidant concentrations, among others, of serum carotenoids between AD and people with cognitive impairment. AD patients had significantly lower α-carotene levels (α-carotene levels were 0.03 moles/L lower in cases versus the controls (95% CI: −0.05,–0.01; *p* = 0.002)), β-carotene levels (beta-carotene levels were 0.11 moles/L lower in cases versus the controls (95% CI:–0.17, –0.04; *p* = 0.0008;), lycopene levels (0.15 moles/L lower levels of lycopene (95% CI: –0.27, –0.02; *p* = 0.02), and lutein levels (pooled mean differences (PMD) –0.13, 95% CI: –0.23, –0.03, *p* = 0.01) in plasma. β-cryptoxanthin levels and zeaxanthin in plasma did not differ between the compared groups.

The above meta-analyses have many limitations, as they are characterized by a high level of statistical heterogeneity, which results from differences in the studied populations (gender, age, and ethnicity) or different study protocols. The strength of the study is the large number of participants and the systematic research methodology.

## 5. Conclusions

The collected data indicate that it cannot be ruled out that carotenoids, both those obtained from the diet and those from supplements, can reduce oxidative stress, inflammation, and Aβ fibril aggregation. Therefore, they are useful in reducing the symptoms and the risk of developing AD and dementia. Despite so much evidence, it is surprising that there is no pharmaceutical formulation dedicated to AD therapy containing carotenoids. Most observational studies confirm that higher intake is associated with increased levels of carotenoids in the serum and brain and, consequently, less neuropathy and cognitive dysfunction. The lack of such a relationship is rarely observed, although observational data from long-term studies may be questionable because they require strict dietary control and are often obtained from interviews with families. While the evaluation of carotenoids in one’s diet may be estimated with low accuracy, analytical determinations of carotenoid levels in the blood are a good measure of one’s intake. On the other hand, a high intake may not guarantee sufficient saturation of carotenoids in the brain due to a low HDL concentration. The results of studies on animal models should also be interpreted with great caution, as they are often performed on animals with induced brain damage and not de facto AD patients. Despite this, the presented reports reinforce the idea of the beneficial antioxidant, anti-inflammatory, and anti-amyloidogenic effects of carotenoids. So far, most reports confirming the neuroprotective activity of carotenoids have been prepared for lycopene, lutein, and zeaxanthin. With the number of natural carotenoids exceeding one thousand, future research should explore this undiscovered area in search of stable, bioavailable ones that maintain cognitive performance.

## Figures and Tables

**Table 1:** Common dietary sources of carotenoids (mg/100 g of fresh weight FW or dried weight DW) and their biological activities.

Natural Source	Common Name	Main Carotenoids	Amount	Other Carotenoids	Ref.
β-carotene: enhancing immune function; antioxidant, anti-inflammatory, and anticancer					[125]
*Daucus carota*	carrot	β-carotene	6.1–8.3 FW	α-carotene, lutein	[126]
*Prunus armeniaca*	apricot	β-carotene	3.5 FW	α-carotene, lutein, β-cryptoxanthin, lycopene	[127]
*Capsicum annuum*	pepper	β-carotene	72–130 DW	β-cryptoxanthin, lutein, α-carotene, zeaxanthin, lycopene	[128]
*Mangifera indica*	mango	β-carotene	3.3–5.8 FW	β-cryptoxanthin, violaxanthin	[128]
*Malphigia punicifolia*	acerola	β-karoten	0.1–0.8 FW	β-cryptoxanthin, lutein, violaxanthin, neoxanthin, neochrome, luteoxanthin, auroxanthin, antheraxanthin, mutatoxanthin, β-cryptoxanthin-5,8-epoxide, β-cryptoxanthin-5,6-epoxide	[129]
*Blakeslea trispora* grown with β-ionone addition	filamentous fungus	β-carotene	3000 DW	γ-carotene, β-zeacarotene, lycopene	[130,131]
β-cryptoxanthin: antioxidant, anti-obesity, anti-inflammatory, anti-osteoporosis, and anticancer					[125]
*Citrus* spp.	orange	β-cryptoxanthin	0.7 FW	α-carotene, β-carotene, lutein	[127]
*Diospyros kaki*	persimmon	β-cryptoxanthin	0.06–0.9 FW	β-carotene, lycopene zeaxanthin, lutein, α-carotene, neoxanthin; violaxanthin	[132]
Lycopene: antioxidant, anticancer, anti-inflammatory, neuroprotective, hepatoprotective, antiproliferative, anti-obesity, and anti-diabetic; enhances immunity and cognition; protects bones; and protects against skin disorders					[125]
*Solanum lycopersicum*	tomato	lycopene	4.5 FW, 46 DW	lutein, β-carotene	[126,133]
*Citrullus lanatus*	watermelon	lycopene	1.6–3.5 FW	α-carotene, β-carotene, β-cryptoxanthin, lutein, lycopene	[133]
*Carica papaya*	papaya	lycopene	1.8–4.2 FW	α-Carotene, β-Carotene, β-cryptoxanthin	[133]
*Psidium guajava*	guava	lycopene	3.2–7.0 FW	phytofluene, β-carotene, γ-carotene, β-cryptoxanthin, rubixanthin, cryptoflavin, lutein, neochrome	[133,134]
*Momordica cochinchinensis*	gac fruit	lycopene	164 FW	β-carotene	[126,133]
*Momordica charantia*	bitter melon	lycopene	27.3 FW	zeaxanthin, β-cryptoxanthin, violaxanthin	[133,135]
Zeaxanthin: antioxidant, anti-inflammatory, neuroprotective, hepatoprotective, immunomodulation, and anti-cancer; improves skin conditions					[125]
*Capsicum annuum*	red paprika	zeaxanthin	89–151 DW	lutein	[133]
Astaxanthin: antioxidant, anti-skin cancer, anti-inflammatory, anti-gastric, anti-hepatoprotective, and anti-diabetes; protection from UV rays; cardiovascular prevention; immune response; neuroprotection					[136]
*Haematococcus pluvialis*, *Chlorella zofingiensis*, *Chlorococcum* sp.	green microalga	astaxanthin	3900–5000 DW	β-carotene, canthaxanthin, lutein	[137]
*Phaffia rhodozyma*	yeast	astaxanthin	38.4–720 DW	γ-carotene, phoenicoxanthin, β-zeacarotene, β-carotene, torulene, torularhodin	[138]
Fucoxanthin: reduces oxidative stress, inhibits the proliferation of a variety of cancer cells, promotes weight loss, acts as an antioxidant and anti-inflammatory agent, has anti-fibrotic activity, and interacts with the intestinal flora to protect intestinal health					[139]
*Sargassum binderi*	brown seaweed	fucoxanthin	740 DW	β-carotene, neoxanthin, violaxanthin	[140]
Crocin: anti-inflammatory, anti-depressant, anti-cancer, anti-hypertensive, anti-atherosclerotic, and anti-platelet aggregation; protects against oxidative damage to brain vasculature, renal tissues, nephrons, the heart, and the retina; protects against neurodegenerative disorders such as epilepsy, Parkinson’s, and Alzheimer’s					[141]
*Crocus sativus*	saffron	crocin	30–1100 DW	crocetin, β-carotene, zeaxanthin	[128,142]
Lutein: antioxidant, anti-inflammatory, autophagy, neuroprotective, photoprotective, hepatoprotective, immunomodulation, and anti-carcinogenic					[125]
*Lactuca sativa*	lettuce	lutein	1.25–2.3 FW	β-carotene violaxanthin, lactucaxanthin	[127,133]
*Brassica oleracea*	broccoli	lutein	2.6–10.5 FW	β-carotene, neoxanthin, violaxanthin	[128]
*Oryza sativa*	black rice	lutein	0.714 FW	zeaxanthin, lycopene and β-carotene	[133]
*Curcubita* spp.	pumpkin	lutein	3.3–38 FW	β-carotene, lycopene, zeaxanthin	[128]
*Spinacia oleracea*	spinach	lutein	6.3 FW	β-carotene	[127]
*Zea mays*	sweetcorn	lutein	0.5 FW	zeaxanthin, α-carotene, β-carotene	[127]

**Table 2:** Astaxanthin’s cognitive benefits in humans.

Trial	Dose	Population	Treatment Period	Accompanying Substances	Ref.
clinical trial	2 mg/day	104 patients with MCI	2 months	Bacopa, phosphatidylserine and vitamin E	[318]
clinical trial	12 mg/day	10 older adults with subjective memory complaints	12 weeks	astaxanthin-rich *Haematococcus pluvialis* extract	[319]
double-blind randomized controlled trial	6 or 12 mg/day	96 older people with forgetfulness	12 weeks	astaxantion-rich *Haematococcus pluvialis* extract	[320]
double-blind randomized controlled trial	6 mg/day	21 people with MCI	12 weeks	sesamin (10 mg/day, derived from *Sesamum indicum*); astaxanthin-rich *Haematococcus pluvialis* extract	[321]
double-blind randomized controlled trial	8 mg/day	54 middle-aged adults (aged 45–64)	8 weeks	astaxanthin-rich extract derived from *Paracoccus carotinifaciens*	[322]
randomised, double-blind, placebo-controlled human trial	6 or 12 mg/day	30 adults (15 men and 15 women) aged 50–69	12 weeks	-	[323]

**Table 3:** Studies dedicated to the effects of saffron and crocin on AD.

Population	AD Model	Doses	Results of Treatment	Ref.
Studies on animal models				
adult Wistar rats	STZ-induced AD	15 and 30 mg of crocin/kg; pre-surgery, 3 weeks	Improvement in learning and memory in the passive avoidance test; decline in MDA concentration; and a critical increment in GPx activity	[388]
adult male albino Wistar rats	STZ-induced AD	100 mg/kg, p.o. for 21 days	Improvement in cognitive performance, lowering of MDA, and increase in thiol levels and GPx activity	[402]
adult male albino Wistar rats	Aβ injection in CA1 area of hippocampus	150, 300 and 600 nmol of crocin via IH injection or 30 mg/kg of crocin; IP administration for 20 days	No changes in Beclin-1 and LC3-II/LC3-I ratio (autophagy), reduction in Bax/Bcl-2 and cleaved caspase-3 (apoptosis)	[397]
male Wistar rats	Acrolein at 3 mg/kg/day p.o.	12.5; 25; 50 mg/kg/day of crocin; p.o., or IP for 2 weeks	Decrease in MDA, Aβ, and phospho-tau levels by modulating MAPKs signalling pathways	[403]
male Wistar rats	D- galactose 400 mg/kg/day; SC for 56 days	7.5; 15; or 30 mg/kg/day of crocin; IP administration	Decrease in MDA and CML formation; increase in MAPK pErk/Erk ratio; phosphorylation of Akt protein; nuclear NF-κB p65 expression; and decrease in NF-κB p65, IL-1β, and TNFα	[404]
male Wistar rats	Intracerebrove-ntricular administration of Aβ25–35	40 mg/kg of crocin	Decrease in the apoptotic cell number (*p* < 0.01) and the expression of Bcl2 in PFC and hippo (*p* < 0.01); increase in Bax, Caspase3, GRP78, and CHOP (*p* < 0.01)	[405]
mice	AD induced by D-galactose and aluminum trichloride	5 or 20 mg/kg of crocin	Improvement in cognition and memory abilities in an open field test; reduction in their escape time in the Morris water maze test; reduction in the Aβ1-42 content in their brains; increase in the levels of GPx, SOD, ACH, and ChAT; reduction in the levels of ROS and acetylcholinesterase in serum, cerebral cortex, and hypothalamus; reduction in Aβ1-42 in the hippocampus	[406]
adult male Wistar rats	chronic restraint stress exposure	30 mg/kg of saffron extract; 15 and 30 mg/kg of crocin; injection	Increase in the activities of GPx and SOD and diminishment of plasma corticosterone levels	[392]
male, 3-month-old Wistar rats	scopolamine 0.2 mg/kg	15 mg/kg or 30 mg/kg of crocin	Counteracted delay-dependent recognition memory deficits, attenuated deficits in the radial water maze test	[376]
C57BL/6 (wild-type mice) and 5XFAD mice	5XFAD mutations characterized by Aβ accumulation, inflammatory astrocytes activation, and cognitive decline	(50 mg/kg/day) or (10 mg/kg/day) of *Crocus sativus* extract; crocin (10 mg/kg/day); orally for one month	Reduction in Aβ load, enhancement of Aβ clearance pathways, upregulation of synaptic proteins NEP and ApoE, and reduced neuroinflammation in the brain	[379]
male Wistar rats	trimethyltin chloride injected IP at single dose of 8 mg/kg	25 mg/kg or 50 mg/kg crocin administered IP for 30 days	Decrease in apoptosis, inflammation, Bax, Casp-9, Pt, Aβ40, and TNF-α, IL-1β,−6; increase in Bcl-2, BDNF, and neuronal thickness of hippocampal partitions	[407]
Studies on the cell lines				
the human neuroblastoma SH-SY5Y and rat pheochromocytoma PC12 cell lines	AD neuronal cell culture models (SH-SY5Y overexpressing APP and PC12 expressing hyperphosphorylated tau)	*trans*-crocin-4 or *trans*-crocetin from 0.1 μM to 1 mM for 24 h or 72 h	Reduction in β- and γ-secretases; accumulation of cellular Aβ, total tau, and tau phosphorylation; and suppression the GSK3β and ERK1/2 kinases	[408]
the HT22 cell lines	l-glutamate- damaged HT22 cells	crocin at the dose of 0.5 and 2 µM for 3 h at 37 °C	Increase in HT22 cell viability, decrease in apoptotic rate, improvement in mitochondrial function, reduction in intracellular accumulation of ROS and Ca^2+^, decrease in expression of Bax, Bad, and cleaved caspase-3, and increase in expression levels of B-cell lymphoma (extra large) and phosphorylated protein kinase B	[406]
mouse hippocampal HT22 cells	cells induced by Aβ1-42	crocetin at 1–10 μM	protection against H_2_O_2_-induced cell death, Aβ1-42-induced cell death, and ROS production	[409]
CD14+ monocytes	cells isolated from 22 sporadic AD patients	5, 10, 25, 50, 100 and 150 μM *trans*-crocetin for 24, 48, 72 and 120 h	Improvement in the clearance of Aβ42 through the involvement of cathepsin B	[410]
BV2 mouse microglial cells	LPS (100 ng/mL) was applied to hippocampal cultures	crocin or crocetin at concentration 0–40 µM for 30 min	The inhibition of LPS-induced nitric oxide (NO) release; reduction in tumor necrosis factor-α, interleukin-1β, NF-κB activation, and intracellular ROS; blocking of hippocampal cell death	[411]
Studies on humans				
46 patients	mild to moderate AD	capsule saffron 30 mg/day (15 mg twice per day)/p.o./16-weeks	Better outcome in cognitive function	[412]
54 patients; Iranian clinical trials (IRCT138711051556N1)	mild-to-moderate AD	capsule saffron 30 mg/day (15 mg twice per day)/p.o./22-weeks	Improvement similar to donepezil in the Alzheimer’s Disease Assessment Scale-cognitive subscale and Clinical Dementia Rating Scale-Sums of Boxes scores	[413]
68 patients	moderate to severe AD	saffron extract capsules (30 mg/day) for 12 months	Reduction in cognitive decline comparable with memantine in SCIRS and FAST evaluation;	[414]
:17 patients, 18 controls; single-blind randomized clinical trial	MCI and aMCImd	125 mg of saffron extract/day/12 months	Improvement in MMSE scores (*p* = 0.015), and MRI, EEG, and ERP in specific structural brain domains	[415]

Abbreviations: malondialdehyde (MDA); glutathione peroxidase (GPx); streptozocin (STZ); intracerebroventricular (icv); intra-hippocampal (IH); intra-peritoneal (IP); subcutaneous (SC); per os (p.o.); Severe Cognitive Impairment Rating Scale (SCIRS) and Functional Assessment Staging (FAST); amnesic and multi domain MCI (aMCImd); electroencephalogram (EEG); electroencephalography-derived event-related potentials (ERPs); magnetic resonance imaging (MRI); mitogen-activated protein kinases (MAPKs); expressions of carboxymethyl lysine (CML) protein; superoxide dismutase (SOD), acetylcholine (ACH), and choline acetyltransferase (ChAT); reactive oxygen species (ROS); Escherichia coli lipopolysaccharide (LPS); Mini-Mental State Examination (MMSE); neprilysin (NEP); apolipoprotein E (ApoE); phospho tau protein (Pt); amyloid-beta 40 (Aβ40); tumor necrosis factor (TNF-α).
